# An Overview of Thunderstorm-Associated Asthma Outbreak in Southwest of Iran

**DOI:** 10.1155/2014/504017

**Published:** 2014-06-29

**Authors:** Arash Forouzan, Kambiz Masoumi, Maryam Haddadzadeh Shoushtari, Esmaeil Idani, Fatemeh Tirandaz, Maryam Feli, Mohammad Ali Assarehzadegan, Ali Asgari Darian

**Affiliations:** ^1^Department of Emergency Medicine, Imam Khomeini Hospital, Azadegan Avenue, Ahvaz Jundishapur University of Medical Sciences, P.O. Box 61936-73116, Ahvaz, Iran; ^2^Departement of Internal Medicine, Division of Pulmonology, Imam Khomeini Hospital, Ahvaz Jundishapur University of Medical Sciences, Ahvaz, Iran; ^3^Department of Immunology, Ahvaz Jundishapour University of Medical Sciences, Ahvaz, Iran

## Abstract

The aim of this study was to report the characteristics and treatment strategies of all patients with acute bronchospasm who were presented to the emergency departments of Ahvaz, Iran, following the occurrence of a thunderstorm on November 2, 2013. A total of 2000 patients presenting with asthma attacks triggered by thunderstorm were interviewed and an initial questionnaire was completed for each individual. After twenty days, patients were asked to complete a supplementary questionnaire, but only 800 of them accepted to do so. The majority of subjects was aged 20–40 years (60.5%) and had no history of asthma in most cases (60.0%). The symptoms had started outdoors for 60.0% of the participants. In most patients, the onset of the condition was on November 2. Short-acting *β*_2_-agonist (salbutamol) and aminophylline were the most commonly prescribed medications in the emergency department. Upon the second interview, 85.3% of the patients were still symptomatic. Overall, 63.6% did not have a follow-up visit after hospital discharge, although all of them were referred to the specialist. The findings of the present study suggest that thunderstorm-associated asthma could affect young adults with no gender priority, with or without asthma history, which put a strain on emergency medical services.

## 1. Introduction

Thunderstorm-associated asthma refers to a sudden surge in the number of acute bronchospasm cases following the occurrence of thunderstorms [1–3]. It is not a formal or definite diagnosis of asthma, but it describes patients suffering from respiratory diseases after thunderstorms. Several observational studies have provided evidences for a relationship between thunderstorms and asthma [4–6]. Although the mechanism of this relationship is not clear yet, different climate changes, that is, temperature drop, higher humidity, thunder and lightning, and increased wind can raise the concentration of allergen particulates whose inhalation, particularly during seasons with high levels of allergens, intensifies asthma attacks [7, 8]. Since not all types of storms cause asthma, meteorological and aeroallergens seem to be simultaneously involved in the development of the condition [9, 10].

Thunderstorm asthma epidemics have been reported in various countries including Australia (Melbourne), England (Birmingham and London), Saudi Arabia, Italy, the U.S., and Canada. The patient characteristics, hospital admission, inadequacy of medical resources to deal with the condition, and the annual cycles of asthma epidemics in various geographic regions have been described [5, 7, 11, 12].

To the best of our knowledge, there is no evidence report of thunderstorm-associated asthma in Iran. The present study sought to clarify this phenomenon through describing the characteristics of patients and their management during the days after storm in November 2, 2013, in Ahvaz.

Ahvaz is the largest city and the capital of Khuzestan province in southwest of Iran. Its population was almost 1.5 million based on 2006 census. It is located on the bank of the Karoon River. The city has a desert climate with many sandstorms and dust storms during the summer. According to a survey by the World Health Organization in 2011, it has the world's worst air pollution. Ahvaz is also an industrial city that has petrochemical, silk textile, and sugar production and steel companies.

The findings of this study may contribute to the identification of prevention methods and promotion of regional health care systems.

## 2. Methods

After the thunderstorm in the evening of November 2, 2013, the emergency departments of hospitals in Ahvaz encountered a sudden raise in the number of patients presenting with acute bronchospasm attacks. This descriptive-analytical study was conducted in Ahvaz, between November 2nd and 20th to assess the demographic characteristic of patients and treatment strategies in emergency departments using an initial questionnaire. Besides, we tried to investigate the patient outcome in 20 days using a supplementary questionnaire.

The study was conducted at the emergency departments of nine hospitals, including three university hospitals (one of which was a pulmonary disease subspecialty center) and six non-university hospitals, throughout Ahvaz. We recorded the number of patients presenting with bronchospasm attack, including shortness of breath and wheezing with or without cough during the mentioned days. Patients with initial diagnosis of shortness of breath due to heart diseases and pregnant patients were excluded from the study.

A nurse or a physician interviewed the patients and filled out a questionnaire containing demographic characteristics, history of respiratory diseases, date and time of visit, chief complaint, and history of smoking. Besides, medications administered in the emergency department and need for oxygen were documented. Given the sudden onset of the epidemic, the questionnaire was designed and distributed three days after the first thunderstorm.

Twenty days later, the subjects who had completed the initial questionnaire were phoned and invited to complete a secondary questionnaire to record the presence of symptoms, number of visits to the emergency department, prescribed medications, occupation, and visits to a specialist. The collected data were finally analyzed. The frequencies of different variables were calculated.

## 3. Results

Of a total of 2000 patients who were interviewed initially, 54.4% were female and 60.5% of them were aged 20–40 years old (Table 1).

Almost all patients complained of shortness of breath and had wheezing in lung auscultation; these symptom and signs were accompanied by cough in 45% of participants. Fever and chest pain were two additional symptoms in 2.6% of patients.

**Figure 1:**
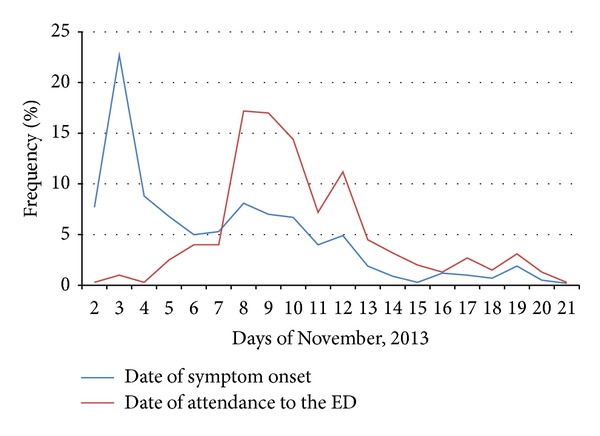
Percentage of patients in regard to date of symptom onset and date of attendance to the emergency department (ED) in November 2013.

Almost 30% of patients experienced their first symptoms on November 2 after a thunderstorm. However, based on the number of primary questionnaires, less than 2% of all 2000 subjects presented to the emergency departments during the first 24 hours of the thunderstorm. The increased number of this respiratory illness patients continued for over three weeks; we calculated the frequency percentage of attending patients to the ED along with symptom onset in each day for almost twenty days (Figure 1).

**Figure 2:**
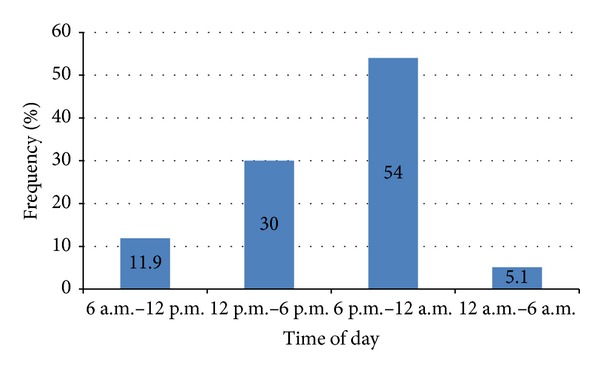
Frequency percentage of patients who attended the emergency department during the 24 hour day.

Over 50% of patients attended the EDs during the evening till midnight (Figure 2).

The past history of a confirmed diagnosis of asthma was positive in 22.7% of patients and 39.2% of patients mentioned some kind of respiratory diseases such as allergies previously. More than 78.2% had no comorbidity or underlying disease. The majority of patients were not smokers (86.8%). Previous history of similar symptoms related to the thunderstorm was positive in 16% of subjects.

Out of 2000 patients, 1800 had provided their contact information, but ultimately only 800 patients accepted to fill out the secondary questionnaire. Based on data collected in the supplementary questionnaire, almost 60% of patients were outdoors at the onset of symptoms.

This study population was asked about their job, 25% of them were housewives and 11.3% were industrial workers who were predisposed to different industrial pollen. The other 63.7% had jobs without any relation to specific pollen.

The mean relapse time of symptoms during the studied period in patients who complete the secondary questionnaire was 2.37 (SD, 2.2), which leads them to return to the ED. The mean number of days off work was 3.9 (SD, 5.4) in our study based on secondary questionnaire.

When filling the second questionnaire, 14.7% of the patients had no symptoms while 60.0%, 7.8%, and 35.6% were still suffering from shortness of breath, coughs, and a combination of coughs, shortness of breath, and wheezing, respectively.

Upon the completion of the secondary questionnaire, 32.8% of the respondents were not using any medications. Others were receiving salbutamol by the inhaled route (27.4%), corticosteroids by the inhaled and oral routes (8.1% and 9.6%, resp.), theophylline (9.5%), and oral salbutamol (0.5%).

Based on primary questionnaire, oxygen was administered to 83.1% of patients. The most frequent drug administered in the emergency department was short-acting *β*_2_-agonist (Table 2).

More than 94% of patients affirmed that they had been prescribed some sort of medication at the time of hospital discharge. While almost all patients were referred to pulmonary clinics, only 36.7% of them had been actually visited by a pulmonologist.

## 4. Discussion

Thunderstorm-associated asthma outbreaks threaten the capacity of health services. Therefore, it is of interest to gather evidence on the affected patient characteristics and design a systematic multidisciplinary approach to such events in all hospitals.

During the mentioned asthma epidemic in Ahvaz, most patients were aged 20–40 years. Studies in other countries have similarly reported thunderstorm to trigger asthma attacks mostly in young people [5, 13–15]. A study in Canada calculated the mean age of the patients as 31 years. It also reported higher prevalence of the disease among men and attributed it to their jobs and their outdoor presence [5]. In the current study in Ahvaz, 25% of the participants were housewives and in the case of gender distribution, 54.9% of patients were female.

The number of patients presenting with intensified asthma attacks in Ahvaz epidemic seemed to be higher than that in similar epidemics in other countries. In fact, 26, 640, 4, and 20 cases were reported in 1983 and 1997 epidemics in England and 2004 and 2010 epidemics in Italy, respectively [15]. Such wide-ranging event, which considerably affects all hospitals in the area, should be investigated comprehensively concerning ethological factors. This could result in considering an early warning system for public and health services in comparable situations.

The thunderstorm with rain occurred on the evening of November 2. Based on our recorded data from the secondary questionnaire, more than 30% of affected patients experienced their first attack in the first 24 hours of the thunderstorm, but less than 2% of primary questionnaires were filled out and documented during the mentioned period. This could result from two reasons; first, the outbreak happened suddenly and during the first day, there was no questionnaire available in the hospitals. The second reason is that some patients underestimated their symptoms and presented to the emergency departments with one or more days delay. Hence, it seems that the number of affected patients was higher than that documented.

In the majority of the patients (60.0%), the symptoms initiated outdoors. Comparable results mentioned in previous studies suggest exposure to high volumes of allergen particulates could be responsible for the attack [15].

Short-acting *β*_2_-agonists, corticosteroids, or methylxanthines were prescribed for more than 94% of patients once discharged from the emergency department. At the time of completing the secondary questionnaire, 88% of subjects were using medications and others discontinued them, but only 14.7% of respondents were symptom-free. Considering these findings, further evaluation of subjects in regard to underlying obstructive lung disease or potential allergy to different pollens could be useful for better understanding of this phenomenon. Moreover, as it is stated in the previous studies, patients who experienced an episode of asthma attack in relation to thunderstorm could be at the risk of relapse even if receiving medications [1].

The administration of aminophylline (which is not the first-line treatment for bronchospasm) in 50.0% of the patients, especially in non-university hospitals, emphasizes the need for retraining regional physicians in regard to the treatment of acute bronchospasm.

The mean number of days off work (3.9 days in one month) indicates the extent of financial burden imposed on the community by this epidemic. An Australian study in 1970 reported the number of days off work due to asthma attacks triggered by thunderstorm to be significantly greater than the rates caused by other types of asthma attacks [16].

In the present study, the majority of the patients were nonsmokers (86.8%). However, we did not have smoker proportion of at-risk population in Ahvaz and could not consider smoking as a protective factor for thunderstorm-associated asthma; but a study in 1997 in Australia argued that smoking prevents the obstruction of the airways following thunderstorms through an unknown mechanism [7].

Finally, 51.7% of our patients had no history of respiratory diseases and had experienced shortness of breath for the first time. This is in line with the epidemic in Saudi Arabia [9]. In a study in Australia, 36% of patients did not have a confirmed diagnosis of asthma and 29% of them reported increased airway hyperresponsiveness experience [7].

In the present study, the major limitation was the lack of a comprehensive patient's registration system in the area. Besides, there are no clear data about the number of daily visits of asthmatic patients in the area before the epidemic. Thus, we could not compare the number of patients before, after, and during the epidemic.

## 5. Conclusion

The findings of the present study suggest that thunderstorm-associated asthma could affect young adults with or without asthma history. Since it is a debilitating phenomenon with high impact on health care services and the whole community, more retrospective and prospective mixed-method studies, such as aerobiological and meteorological ones, should be designed for better recognizing of the phenomenon.

## Figures and Tables

**Table 1:** The number and frequency percentage of affected patients based on their age category.

Age	<20	20–40	40–60	>60
Numbers (percentage)	186 (11.3%)	1486 (60.5%)	397 (24%)	68 (4.2%)

**Table 2:** Frequency percentage of administering drugs in the emergency department of nine studied hospitals.

Drugs	Short-acting *β*_2_-agonist	Corticosteroid injection	Oral corticosteroid	Aminophylline infusion
Percentage	63.1%	40%	13.5%	50%
